# Supplementary material for: Initiation phase cellular reprogramming ameliorates DNA damage in the ERCC1 mouse model of premature aging
Source: Front Aging. 2024 Jan 23;4:1323194. doi: 10.3389/fragi.2023.1323194 (PMC10844398; doi:10.3389/fragi.2023.1323194)
Supplement: Supplementary file 1 [file Presentation1.pdf]

Supplementary Material

Supplementary Figures

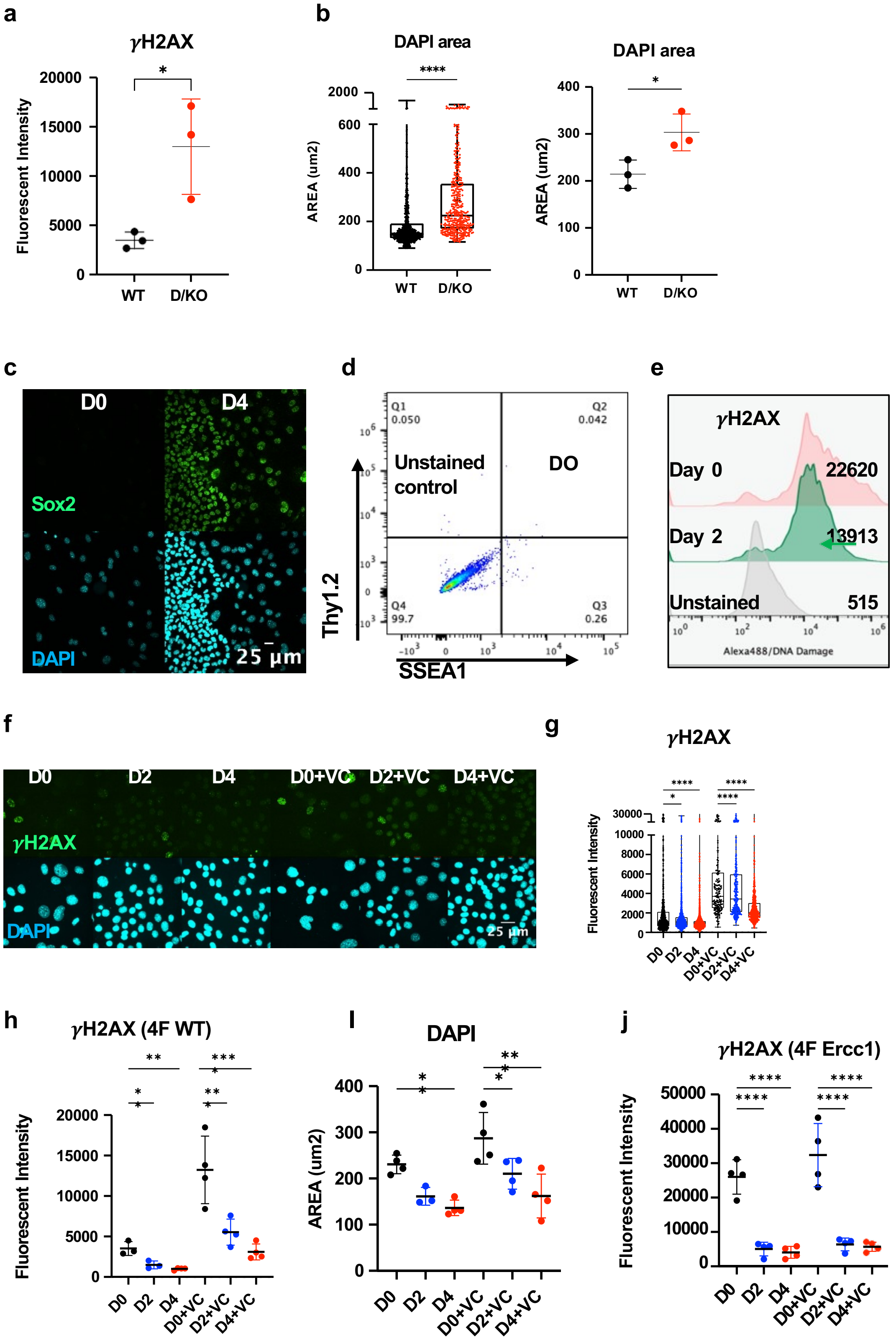

Fig. S1

Figure S1: Initiation phase reprogramming in 4Fj$^{+/-}$ rtTA$^{+/-}$ Ercc1$^{\Delta/-}$ accelerated aging model promotes DNA damage repair.
(a) Quantification of γH2AX IF from 3 experiments in fibroblasts from wildtype (WT) and Ercc1$^{\Delta/-}$ (D/KO) mice, imaged with Nikon laser confocal spinning disc, according to unpaired t-test, * p<0.05. **(b)**. Quantification of DAPI in fibroblasts from wildtype (WT) and Ercc1$^{\Delta/-}$ (D/KO) mice, imaged with Nikon laser confocal spinning disc, according to Mann-Whitney test, **** p<0.0001. Quantification of DAPI area from 3 experiments in fibroblasts from wildtype (WT) and Ercc1$^{\Delta/-}$ (D/KO) mice, imaged with Nikon laser confocal spinning disc, according to unpaired t-test, * p<0.05 **(c)** IF image of Sox2 and DAPI in 4F.D/KO Fbs after 4 days doxycycline induction, 40x **(d)** FACS unstained control for analysis of reprogramming Fbs markers Thy1.2 and SSEA1. **(e)** FACS analysis γH2AX levels following reprogramming of 4F.D/KO Fbs at day 0, day 2, and unstained control. **(f)** IF images of γH2AX and DAPI during representative time course analysis of 4F.WT Fb +/-VC at 100x. **(g)** Quantification of γH2AX images during time course shows significantly decreased levels at day 2 and day 4 according to one way ANOVA, **** p<0.0001. **(h)** Quantification of mean yH2AX levels in 4F WT from 3 or 4 experiments during time course shows significant decrease to levels at day 2 and day 4 according to one way ANOVA, ** p<0.01. **(i)** Quantification of DAPI area during time course shows decrease in size at day 2 and day 4 based on mean values of 4 experiments according to one way ANOVA, *** p<0.001, ** p<0.01, *p<0.05. **(j)** Quantification of mean γH2AX levels in 4F D/KO from 4 experiments during time course shows significant decrease to levels at day 2 and day 4 according to one way ANOVA, ** p<0.0001.

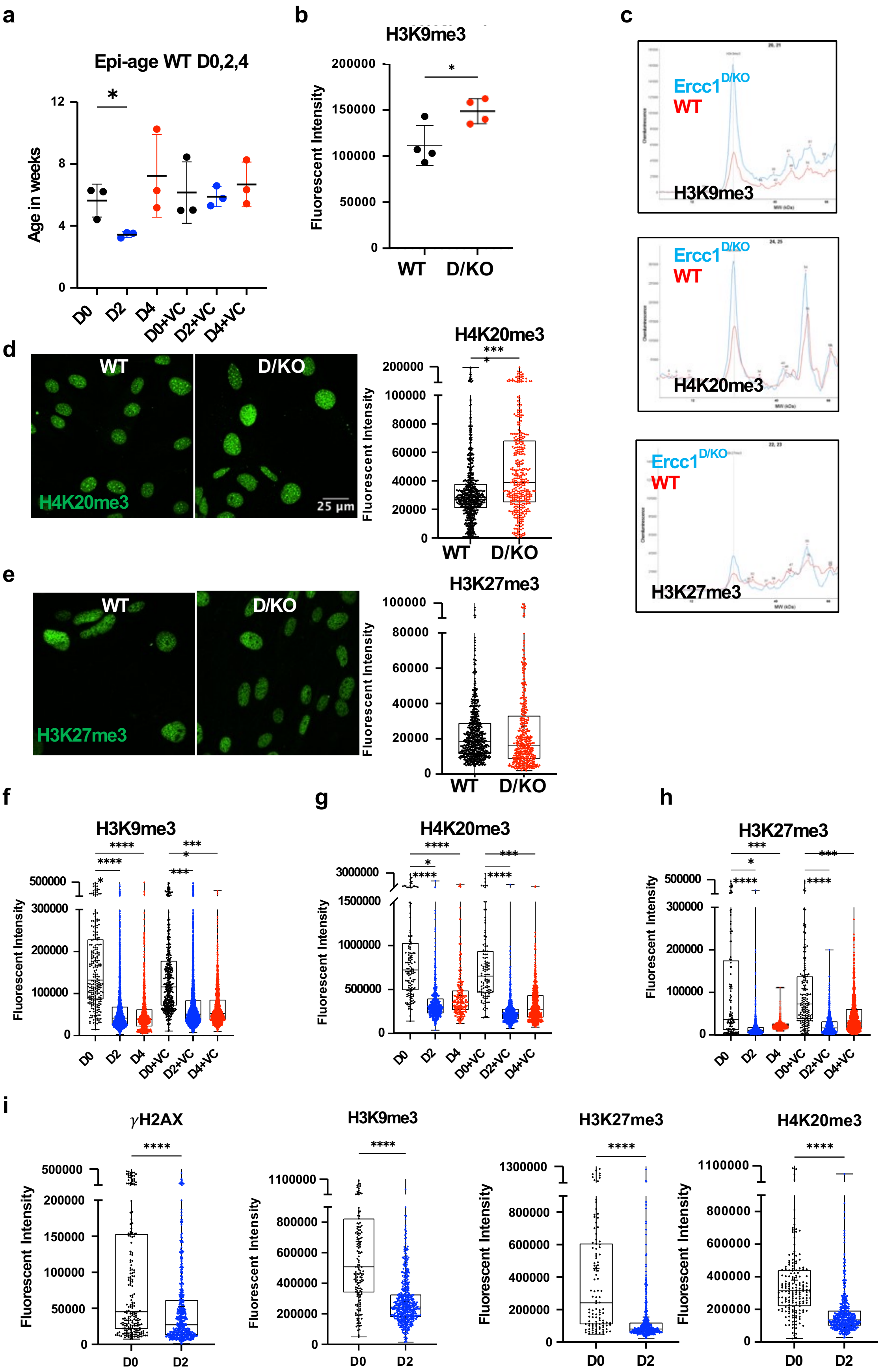
a
Epi-age WT D0,2,4
Age in weeks
D0
D2
D4
D0+VC
D2+VC
D4+VC
b
H3K9me3
Fluorescent Intensity
WT
D/KO
c
Ercc1 D/KO
WT
H3K9me3
H4K20me3
H3K27me3
d
WT
D/KO
H4K20me3
25 μm
e
H3K27me3
f
H3K9me3
g
H4K20me3
h
H3K27me3
i
γH2AX
H3K9me3
H3K27me3
H4K20me3

Fig. S2

Figure S2: DNA methylation clock is restored in Ercc1$^{\Delta/-}$ following short term reprogramming.
(a) DNA Methylation Age Skin Final clock analysis of WT Fb during time course, n = 3, * p<0.05 according to unpaired t-test. **(b)** Quantification of mean values of H3K9me3 levels in fibroblasts from WT and D/KO mice (n=4) according to unpaired t-test, * p<0.05. (**c)** Western capillary chemiluminescence quantification shows increased H3K9me3 and H4K20me3, with unchanged H3K27me3 in fibroblasts from D/KO vs WT mice (n=1 each). **(d)** IF and quantification shows increased H4K20me3 in fibroblasts from D/KO mice vs WT, imaged with Nikon laser confocal spinning disc, according to Mann-Whitney test, **** p<0.0001 **(e)** IF quantification shows unchaged H3K27me3 in fibroblasts from WT and D/KO mice, imaged with Nikon laser confocal spinning disc. **(f, g,h)** IF quantification of 2nd experiment shows significantly decreased H3K9me3, H4K20me3, and H3K27me3 levels during time course in 4F.D/KO Fbs after 2 and 4 days doxycycline induction with or without VC at 100x according to Kruskal-Wallis test, * p<0.000. **(i)** IF quantification of 3rd experiment shows γH2AX, H3K9me3, H4K20me3, and H3K27me3 in 4F.D/KO Fbs after 2 doxycycline induction at 100x according to Mann-Whitney test, * p<0.000.

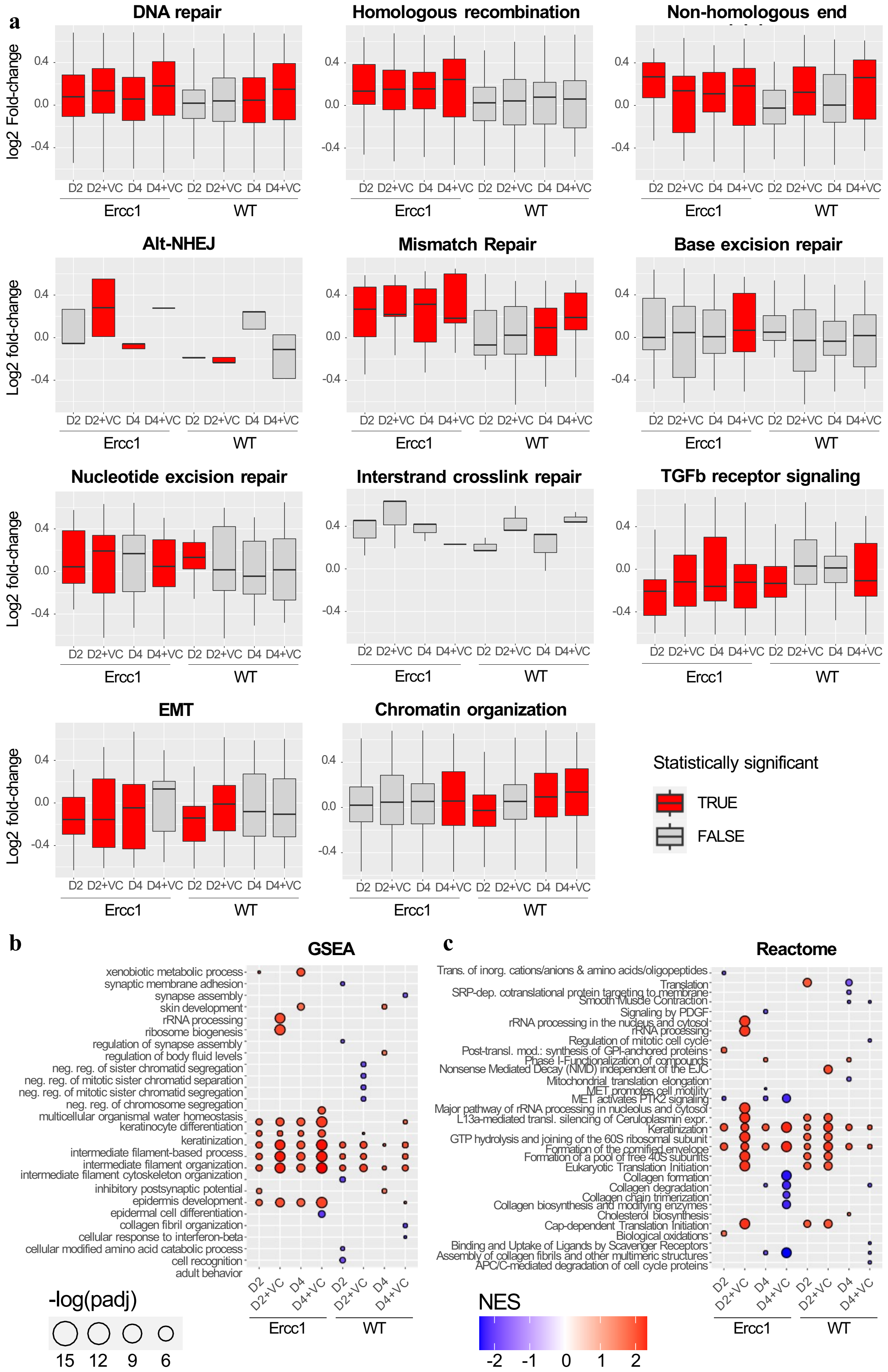
a
DNA repair
Homologous recombination
Non-homologous end
Alt-NHEJ
Mismatch Repair
Base excision repair
Nucleotide excision repair
Interstrand crosslink repair
TGFb receptor signaling
EMT
Chromatin organization
log2 Fold-change
Log2 fold-change
0.4
0.0
-0.4
D2 D2+VC D4 D4+VC D2 D2+VC D4 D4+VC
Ercc1
WT
Statistically significant
TRUE
FALSE
b
GSEA
xenobiotic metabolic process
synaptic membrane adhesion
synapse assembly
skin development
rRNA processing
ribosome biogenesis
regulation of synapse assembly
regulation of body fluid levels
neg. reg. of sister chromatid segregation
neg. reg. of mitotic sister chromatid separation
neg. reg. of mitotic sister chromatid segregation
neg. reg. of chromosome segregation
multicellular organismal water homeostasis
keratinocyte differentiation
keratinization
intermediate filament-based process
intermediate filament organization
intermediate filament cytoskeleton organization
inhibitory postsynaptic potential
epidermis development
epidermal cell differentiation
collagen fibril organization
cellular response to interferon-beta
cellular modified amino acid catabolic process
cell recognition
adult behavior
-log(padj)
15 12 9 6
c
Reactome
Trans. of inorg. cations/anions & amino acids/oligopeptides
Translation
SRP-dep. cotranslational protein targeting to membrane
Smooth Muscle Contraction
Signaling by PDGF
rRNA processing in the nucleus and cytosol
rRNA processing
Regulation of mitotic cell cycle
Post-transl. mod.: synthesis of GPI-anchored proteins
Phase I-Functionalization of compounds
Nonsense Mediated Decay (NMD) independent of the EJC
Mitochondrial translation elongation
MET promotes cell motility
MET activates PTK2 signaling
Major pathway of rRNA processing in nucleolus and cytosol
L13a-mediated transl. silencing of Ceruloplasmin expr.
Keratinization
GTP hydrolysis and joining of the 60S ribosomal subunit
Formation of the cornified envelope
Formation of a pool of free 40S subunits
Eukaryotic Translation Initiation
Collagen formation
Collagen degradation
Collagen chain trimerization
Collagen biosynthesis and modifying enzymes
Cholesterol biosynthesis
Cap-dependent Translation Initiation
Biological oxidations
Binding and Uptake of Ligands by Scavenger Receptors
Assembly of collagen fibrils and other multimeric structures
APC/C-mediated degradation of cell cycle proteins
NES
-2 -1 0 1 2

Fig. S3

Figure S3: Initation phase reprogramming drives transcriptomic reset and upregulates DNA repair and chromatin organization in Ercc1$^{\Delta/-}$

(a) Log2 fold-change of genes within a given GO pathway for Ercc1 and WT reprogramming at days 0, 2, and 4 with and without VC enhancement. The inner boxplot depicts medians and the first and third quartiles, with whiskers extending up to the 1.5x interquartile range and outliers removed for improved visualization of differences between conditions. Statistical significance (Wilcoxon Test, p-value < 0.05) is indicated by red coloring. **(b and c)** GSEA and Reactome analysis of Ercc1 and WT reprogramming considering log2 fold-changes between day 0 and day 2 of reprogramming and day 0 and day 4 of reprogramming with and without VC enhancement. Gene ontology biological process terms and Reactome terms are plotted against the normalized enrichment score (NES) with -log(adjusted p-value) illustrated through circle size. The top 10 terms for each condition are included.

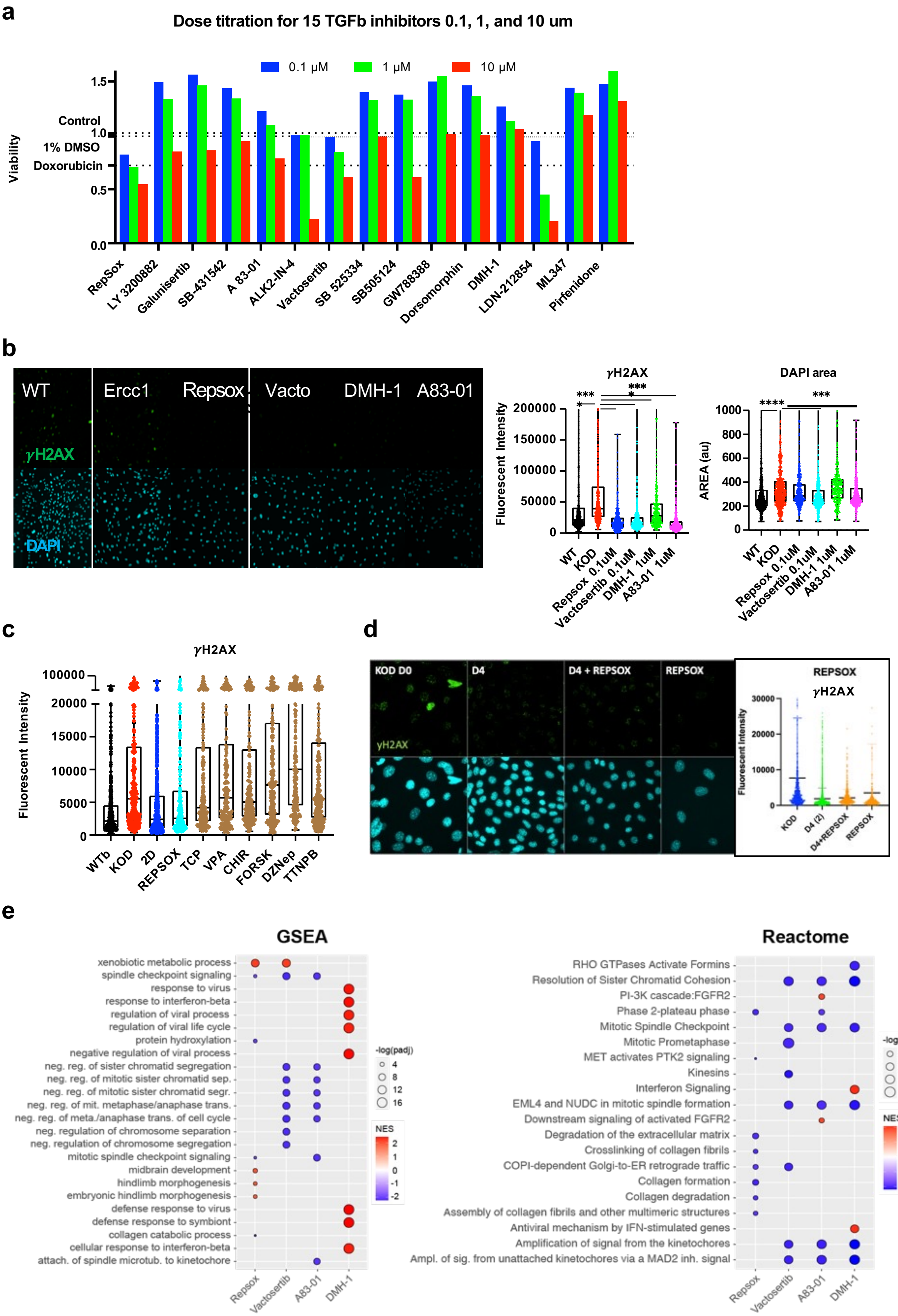
a
Dose titration for 15 TGFb inhibitors 0.1, 1, and 10 um
0.1 µM
1 µM
10 µM
Viability
Control
1% DMSO
Doxorubicin
RepSox
LY 3200882
Galunisertib
SB-431542
A 83-01
ALK2-IN-4
Vactosertib
SB 525334
SB505124
GW788388
Dorsomorphin
DMH-1
LDN-212854
ML347
Pirfenidone
b
WT
Ercc1
Repsox
Vacto
DMH-1
A83-01
γH2AX
DAPI
Fluorescent Intensity
DAPI area
AREA (au)
Repsox 0.1uM
Vactosertib 0.1uM
DMH-1 1uM
A83-01 1uM
c
γH2AX
Fluorescent Intensity
WTb
KOD
2D
REPSOX
TCP
VPA
CHIR
FORSK
DZNep
TTNPB
d
KOD D0
D4
D4 + REPSOX
REPSOX
REPSOX
γH2AX
e
GSEA
Reactome
xenobiotic metabolic process
spindle checkpoint signaling
response to virus
response to interferon-beta
regulation of viral process
regulation of viral life cycle
protein hydroxylation
negative regulation of viral process
neg. reg. of sister chromatid segregation
neg. reg. of mitotic sister chromatid sep.
neg. reg. of mitotic sister chromatid segr.
neg. reg. of mit. metaphase/anaphase trans.
neg. reg. of meta./anaphase trans. of cell cycle
neg. regulation of chromosome separation
neg. regulation of chromosome segregation
mitotic spindle checkpoint signaling
midbrain development
hindlimb morphogenesis
embryonic hindlimb morphogenesis
defense response to virus
defense response to symbiont
collagen catabolic process
cellular response to interferon-beta
attach. of spindle microtub. to kinetochore
RHO GTPases Activate Formins
Resolution of Sister Chromatid Cohesion
PI-3K cascade:FGFR2
Phase 2-plateau phase
Mitotic Spindle Checkpoint
Mitotic Prometaphase
MET activates PTK2 signaling
Kinesins
Interferon Signaling
EML4 and NUDC in mitotic spindle formation
Downstream signaling of activated FGFR2
Degradation of the extracellular matrix
Crosslinking of collagen fibrils
COPI-dependent Golgi-to-ER retrograde traffic
Collagen formation
Collagen degradation
Assembly of collagen fibrils and other multimeric structures
Antiviral mechanism by IFN-stimulated genes
Amplification of signal from the kinetochores
Ampl. of sig. from unattached kinetochores via a MAD2 inh. signal
-log(padj)
NES
Repsox
Vactosertib
A83-01
DMH-1

Fig. S4

Figure S4: Inhibition of ALK5 or ALK2 receptors improves DNA damage phenotype and resets the DNA methylation clock and transcriptome in Ercc1$^{\Delta/-}$

(a) Cell viability quantification using MTS assay in 96 well plates following treatment with high, medium, and low concentrations of 15 inhibitors in triplicate. Dosage range is 10um, 1um, and 0.1 um. Controls are represented by dashed lines and include untreated D/KO Fbs, 1% DMSO, and Doxorubicin (0.1um). **(b)** IF images and quantification of γH2AX levels and DAPI area in WT vs D/KO Fbs treated with Repsox, Vactosertib, DMH-1, and A83-01 in 24 wells plates, n = 1. **(c)** IF quantification of yH2AX levels comparing 2 days of doxycline induction to seven single small molecules used in chemical reprogramming at published dosages: Repsox (3um), TCP (10um), VPA (500um), CHIR99021 (5um), Forskolin (10um), DZNep (0.5um), and TTNPB (1um), n = 1. **(d)** IF images and quantification of γH2AX levels in 4F.D/KO cells after 4 days of induction either with doxycycline alone, doxycycline and Repsox, or Repsox alone, n = 1. **(e)** GSEA and Reactome analysis of Ercc1 cells treated with TGFb inhibitors considering log2 fold-changes, n = 3. Gene ontology biological process terms or Reactome terms are plotted against the normalized enrichment score (NES) with -log(adjusted p-value) indicated through circle size. The top 10 terms for each condition are included.

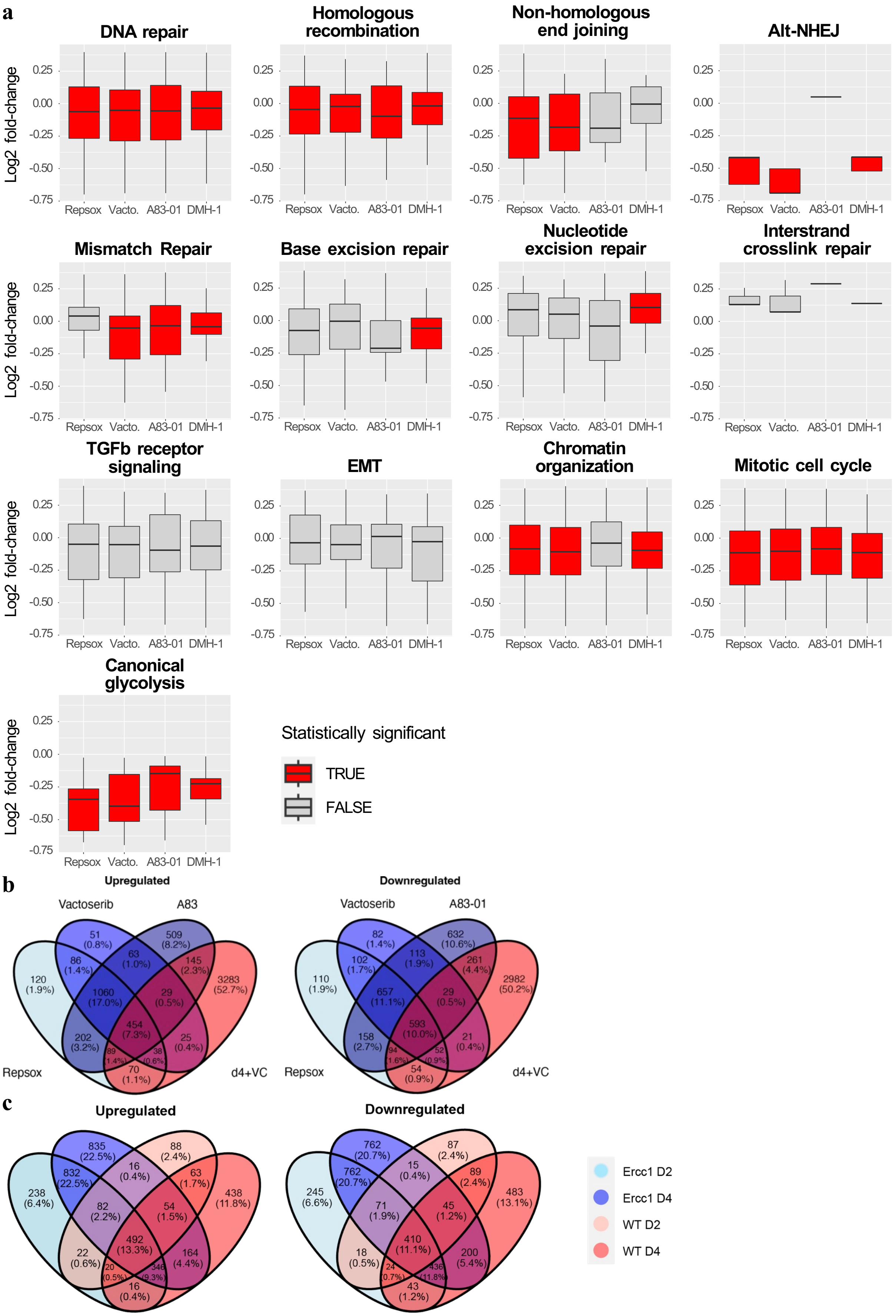
a
DNA repair
Homologous recombination
Non-homologous end joining
Alt-NHEJ
Mismatch Repair
Base excision repair
Nucleotide excision repair
Interstrand crosslink repair
TGFb receptor signaling
EMT
Chromatin organization
Mitotic cell cycle
Canonical glycolysis
Log2 fold-change
Repsox
Vacto.
A83-01
DMH-1
Statistically significant
TRUE
FALSE
b
Upregulated
Downregulated
Vactoserib
A83
A83-01
Repsox
d4+VC
c
Upregulated
Downregulated
Ercc1 D2
Ercc1 D4
WT D2
WT D4

Fig. S5

Figure S5: GO term analysis of TGFb inhibition in Ercc1$^{\Delta/-}$. (a) Log2 fold-change of genes within a given GO pathway for Ercc1 cells treated with TGFb inhibitors compared to non-treated Ercc1 cells. The inner boxplot depicts medians and the first and third quartiles, with whiskers extending up to the 1.5x interquartile range and outliers removed for improved visualization of differences between conditions. Statistical significance (Wilcoxon Test, p-value < 0.05) is indicated by red coloring. **(b)** Venn Diagram showing the overlap of significant genes (adjusted p-value < 0.05) for the ALK5 inhibitors and enhanced reprogramming day 4+VC. **(c)** Venn Diagram showing the overlap of significant genes (adjusted p-value < 0.05) for the reprogramming time course at day 2 and 4 without VC.
